# Profiles of Human Serum Antibody Responses Elicited by Three Leading HIV Vaccines Focusing on the Induction of Env-Specific Antibodies

**DOI:** 10.1371/journal.pone.0013916

**Published:** 2010-11-09

**Authors:** Michael Vaine, Shixia Wang, Qin Liu, James Arthos, David Montefiori, Paul Goepfert, M. Juliana McElrath, Shan Lu

**Affiliations:** 1 Laboratory of Nucleic Acid Vaccines, Department of Medicine, University of Massachusetts Medical School, Worcester, Massachusetts, United States of America; 2 Division of Preventive and Behavior Medicine, Department of Medicine, University of Massachusetts Medical School, Worcester, Massachusetts, United States of America; 3 Laboratory of Immunoregulation, National Institute of Allergy and Infectious Diseases, National Institutes of Health, Bethesda, Maryland, United States of America; 4 Duke University Medical Center, Durham, North Carolina, United States of America; 5 Department of Medicine, University of Alabama at Birmingham, Birmingham, Alabama, United States of America; 6 Department of Microbiology, University of Alabama at Birmingham, Birmingham, Alabama, United States of America; 7 Department of Medicine, University of Washington School of Medicine, Seattle, Washington, United States of America; 8 Laboratory Medicine, University of Washington School of Medicine, Seattle, Washington, United States of America; 9 Vaccine and Infectious Disease Institute, Fred Hutchinson Cancer Research Center, Seattle, Washington, United States of America; University of California San Francisco, United States of America

## Abstract

In the current report, we compared the specificities of antibody responses in sera from volunteers enrolled in three US NIH-supported HIV vaccine trials using different immunization regimens. HIV-1 Env-specific binding antibody, neutralizing antibody, antibody-dependent cell-mediated cytotoxicity (ADCC), and profiles of antibody specificity were analyzed for human immune sera collected from vaccinees enrolled in the NIH HIV Vaccine Trial Network (HVTN) Study #041 (recombinant protein alone), HVTN Study #203 (poxviral vector prime-protein boost), and the DP6-001 study (DNA prime-protein boost). Vaccinees from HVTN Study #041 had the highest neutralizing antibody activities against the sensitive virus along with the highest binding antibody responses, particularly those directed toward the V3 loop. DP6-001 sera showed a higher frequency of positive neutralizing antibody activities against more resistant viral isolate with a significantly higher CD4 binding site (CD4bs) antibody response compared to both HVTN studies #041 and #203. No differences were found in CD4-induced (CD4i) antibody responses, ADCC activity, or complement activation by Env-specific antibody among these sera. Given recent renewed interest in realizing the importance of antibody responses for next generation HIV vaccine development, different antibody profiles shown in the current report, based on the analysis of a wide range of antibody parameters, provide critical biomarker information for the selection of HIV vaccines for more advanced human studies and, in particular, those that can elicit antibodies targeting conformational-sensitive and functionally conserved epitopes.

## Introduction

Developing a safe and effective vaccine to control the global transmission of Human Immunodeficiency Virus Type 1 (HIV-1) remains one of the greatest challenges. The surprising outcome of the STEP trial [1] demonstrated the danger of relying on one type of vaccine and not paying equal attention to other vaccination approaches [2]–[3]. Passive protection studies using neutralizing monoclonal antibodies (mAbs) have demonstrated the utility of antibodies in controlling infection in non-human primates [4], [5], [6], [7], [8], [9], [10]. Furthermore, recently completed Phase III human HIV-1 vaccine trial, RV144, using a canarypox vector prime-recombinant envelope (Env) protein boost design, showed a low but significant 31% reduction of infection compared with placebo [11]. The mechanism for such protection in RV144 is unknown but protective antibody is suspected to play a key role. However, in-depth analysis of antibody responses elicited in RV144 trial volunteers requires baseline information on the qualities of human anti-Env antibody responses elicited by other types of HIV-1 vaccines. Currently, such comparative analysis is lacking in the literature. Recently, several new vaccination approaches have significantly improved the magnitude or quality of HIV-1 Env-specific antibody responses in humans and, thus, provide the opportunity to compare the unique profiles of antibody responses elicited by different HIV vaccine strategies.

In the current report, human vaccinee sera from three HIV-1 vaccine studies using different immunization approaches (Table 1) were analyzed for the relative levels of binding and neutralizing antibodies, the fine specificities of antibodies present in each serum, and the ability to mediate other potentially protective processes, including complement activation and Antibody-Dependent Cell-mediated Cytoxicity (ADCC). Our results indicated that each HIV vaccine regimen can elicit unique profile of antibody responses. This finding will be very useful to improve the design of HIV vaccines to elicit the optimal protective antibody responses in humans.

**Table 1:** Summary of vaccine regimens.

Trial	Prime Immunizations			Boost Immunizations			HIV-1 strains	Adjuvant
	Type	Dose	Weeks	Type	Dose (µg)	Weeks		
HVTN 041	N/A	N/A	N/A	gp120 protein	5, 20, or 100	0, 4, 12	W61D	AS02_A_ #
HVTN 203	Canarypox	10^7.26^ TCID50	0, 4, 12, 24	gp120 protein	600	12, 24	MN, GNE8	Alum
DP6-001	DNA	1.2 mg	0, 4, 12	gp120 protein	375	20, 28	A, B, Bal, C, E*	QS21

# QS-21 & 3D-MPL in o/w emulsion.

*A: 92UG037 B: 92US715 Bal: Ba-L C:96ZM651 E: 93TH976.

## Results

All three candidate HIV vaccines included in the current analysis were designed to elicit HIV-1 Env-specific antibody responses (Table 1). HVTN 203 was an early phase clinical study using a canarypox prime-protein boost regimen prior to the full-scale RV144 efficacy trial. Volunteers from HVTN203 (Group B) received the canarypox vector expressing a clade B Env, and were boosted with a bivalent clade B/B Env protein formulation from HIV-1 isolates, MN, and GNE8 [12], whereas RV144 expressed a clade E Env by canarypox vector, which was then boosted with bivalent clade B/E Env proteins [11]. Volunteers in the HVTN 203 trial received a total of four canarypox vector immunizations in addition to two protein boosts adjuvanted with alum that were overlapped with the last two canarypox immunizations. Protein boosts consisted of the same recombinant Env protein vaccine that failed to show protective efficacy in a Phase III clinical trial when used alone [13]. HVTN 041 tested the immunogenicity of recombinant Env protein derived from the HIV-1 isolate W61D, adjuvanted in AS02_A_, without any prime immunizations [14]. The DP6-001 trial used a DNA prime-recombinant protein boost immunization approach delivering a 5-valent Env formulation from HIV-1 isolates of clades A, B, C, and E [15]. Human volunteers were first immunized three times with Env-expressing DNA vaccines, followed by two boosts using matched recombinant Env proteins (gp120) in QS-21 adjuvant.

Neutralizing antibody activity has been a key parameter in HIV vaccine research to measure the protective potential of immune sera specific for HIV-1 Env antigens [16], [17]. Results of neutralizing antibody activities in three sets of sera included in the current report were previously reported and showed diverse profiles [12], [14], [15]. In contrast to sera from the DP6-001 study, which were capable of neutralizing a broad range of T-cell line adapted (TCLA) and primary HIV-1 isolates [15], sera from the HVTN 041 and HVTN 203 studies was only capable of neutralizing autologous and TCLA viral strains [12], [14]. Because previous neutralizing activity analyses from each trial were done in different assay systems, making direct comparisons difficult, a new but limited set of neutralization assays were conducted by using pseudotyped viruses expressing three model HIV-1 primary Env antigens with varying degrees of sensitivity to neutralization to confirm the previously reported neutralizing patterns for these three sets of human sera. No extensive NAb analysis was done in the current study, as they have been done in previously published reports [12], [14], [15].

**Figure 1:**
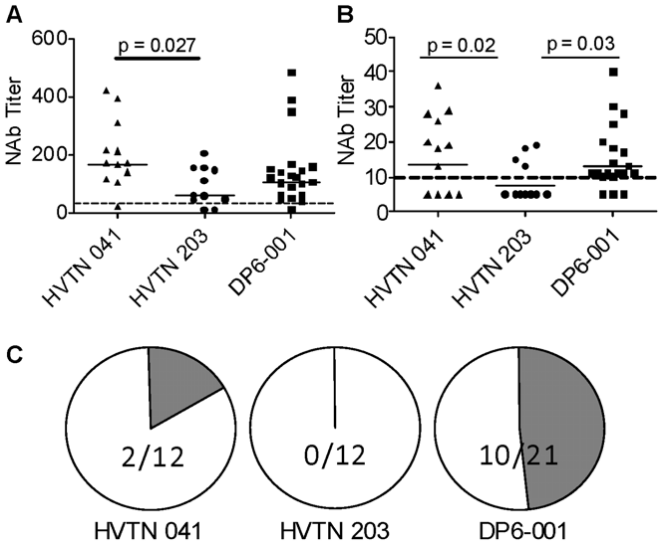
Confirmation of neutralizing activities against representative HIV isolates. Neutralization antibody titers at 50% inhibition for each serum are shown against either SF162 (A) or SS1196.1 (B). Neutralizing activities against SC422661.8 (C) is shown as the fractions of individual sera from each trial either capable of achieving at least 50% inhibition of infection at a 1∶10 serum dilution (shaded portion) or unable to achieve 50% inhibition (open portion). All p values reaching significance (p<0.05) are presented in the figure. All other comparisons did not reach significant based upon Kruskal-Wallis and Dunn'significance tests.

The vast majority of all sera tested, including 11 out of 12 (92%) from the HVTN 041 study, 10 out of 12 sera (83%) from the HVTN 203 study, and 20 out of 21 (95%) from the DP6-001 study, were capable of neutralizing SF162, a primary isolate highly sensitive to neutralization (Fig 1A). Geometric mean ID50 titers were 1∶164 for HVTN 041, 1∶62 for the HVTN 203 trial sera, and 1∶104 for DP6-001. Sera from the HVTN 041 trial were significantly more potent than those from the HVTN 203 study against the sensitive isolate SF162 (p = 0.027), but not significantly different from DP6-001.

Neutralizing activities against SS1196, a primary isolate that is moderately sensitive to neutralization, allowed for some differentiation of the neutralization potential of each trial sera (Fig 1B). Only 4 of the 12 sera (33%) from the HVTN 203 trial were capable of neutralizing SS1196 at a 1∶10 dilution but 8 of the 12 sera (67%) from the HVTN 041 trial were capable of neutralizing this virus. In contrast, 18 of the 21 sera (86%) from the DP6-001 trial were capable of neutralizing SS1196. Both the HVTN 041 and DP6-001 trials elicited higher titers than the HVTN 203 trial (p = 0.02 and p = 0.03, respectively).

**Figure 2:**
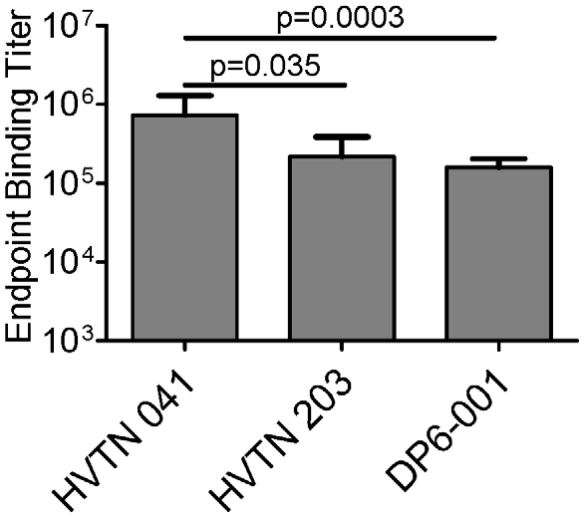
Geometric mean endpoint binding titers of sera from each of three human vaccine trials against a clade B recombinant gp120 protein (JR-FL). Error bars indicate standard error.

The third pseudotyped virus tested in the current analysis expressed Env from the HIV-1 isolate, SC422661.8, a Tier 2 virus representative of those found shortly after the establishment of HIV-1 infection and known to be highly resistant to neutralization [18]. A significant drop of neutralizing activities was observed with sera from all three vaccine trials against this virus (Fig 1C). None of the sera from the HVTN 203 trial were capable of reaching 50% neutralization at the lowest dilution tested (1∶10). Similarly, neutralizing activity against this isolate was only observed in two sera (17%) from the HVTN 041 trial. However, 10 of the 21 sera (48%) from the DP6-001 trial were capable of neutralizing SC422661.8 at a 1∶10 dilution. This occurred despite the fact that, on average, individuals in the DP6-001 had either lower or equivalent titers of Env-specific binding antibodies when compared to other two trial sera (Fig 2 below). The lack of neutralizing activity from the HVTN 203 and 041 trials against more resistant isolates, and the low titer neutralization seen in the samples from the DP6-001 trial are both consistent with previously reported neutralization profiles [12], [14], [15].

In order to understand what features of the antibody responses elicited by each of these sera may be responsible for the difference in their neutralization profiles, a wide spectrum of analyses were conducted to understand the quality of different sera. The first was Env-specific binding antibodies. The gp120 protein from the clade B JR-FL strain was chosen as the model antigen to examine binding titers because it is derived from a well-characterized primary isolate and while each trial tested here was formulated with at least one clade B component, JR-FL was not a component in any of the formulations. Antibody levels generated by the HVTN 041 formulation were found to be significantly higher than the titers of binding antibodies generated in either the HVTN 203 or DP6-001 clinical trials (p = 0.035 and p = 0.0003, respectively) (Fig 2), suggesting that gp120 adjuvanted with AS02_A_ is an exceptionally immunogenic formulation.

**Figure 3:**
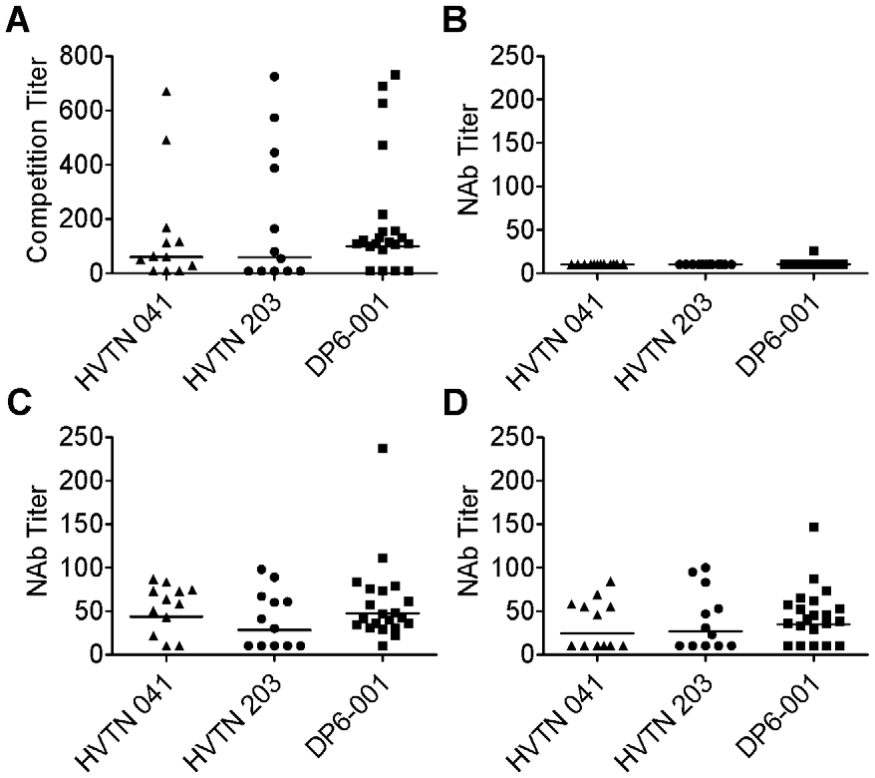
Analysis of antibodies against CD4 inducible (CD4i) epitopes. A) The presence of co-receptor binding site-directed antibodies was assayed by competition with the mAb, 17b. Competition titer indicates the serum dilution capable of outcompeting 50% of pseudoviral binding to 17b. B) Neutralizing antibody titers against HIV-1 JR-FL isolate without sCD4 treatment. C) Neutralizing antibody titers against HIV-1 JR-FL isolate with sCD4 treatment. D) Effect of V3 peptide treatment on neutralizing activity against sCD4-treated JR-FL.

Antibodies directed to CD4 inducible (CD4i) epitopes are frequently elicited in HIV-infected individuals [19] although their role in controlling viral infection is currently unknown. Prior exposure of pseudovirus to soluble CD4 (sCD4) can expose CD4i epitopes, such as the co-receptor binding site, on the viral envelope [20]. Sera from each trial included in the current study were assayed for their ability to outcompete binding to 17b, a mAb that targets the co-receptor binding site. High frequency and titers of 17b-like antibodies were detected in all three vaccine trials (Fig 3A). Seven out of 12 sera (58%) from the HVTN 203 trial, 9 out of 12 (75%) from HVTN 041, and 17 out of 21 (81%) from DP6-001 were able to outcompete binding to 17b. Interestingly, those sera that did compete did so at high titer, indicating an abundance of antibodies with this specificity.

Next assay evaluated if the CD4i antibodies found in the sera are functional in a modified neutralization assay. Pseudotyped viruses expressing Env from the JR-FL isolate were treated with sCD4 prior to incubation with serum. While without prior sCD4 treatment, JR-FL was difficult to neutralize by sera from all three trials (Fig. 3B), significant neutralizing activities against JR-FL Env pseudotyped viruses upon exposure to sCD4 were found in these sera: 7 out of 12 (58%) from HVTN 203, 10 out of 12 (83%) from HVTN 041, and 20 out of 21 (95%) from DP6-001 with positive neutralizing activities (Fig 3C). Geometric mean neutralizing titers for HVTN 203, HVTN 041, and DP6-001 were 1∶28, 1∶44, and 1∶49, respectively. This data suggests that under the proper conditions, CD4i antibodies present in vaccinee sera would be capable of neutralizing heterologous isolates of HIV-1.

Because it has been reported that sCD4 treatment leads to increased exposure of the V3 loop [21], we attempted to determine if the neutralizing activity observed after sCD4 treatment was due to recognition of the V3 loop or recognition of the co-receptor binding site by the 17b-like antibodies detected through competition. Vaccinee immune sera were incubated with a synthetic peptide matched to the V3 loop sequence of the JR-FL Env prior to the exposure of sCD4-treated JR-FL. This resulted in a slight drop in the geometric mean NAb titer of HVTN 203 sera to 26, of HVTN 041 sera to 25, and of DP6-001 sera to 34 (Fig 3D). This drop in potency was also accompanied by a drop in the frequency of positive neutralizing sera to 6 out of 12 sera (50%) in the HVTN 041 trial and to 16 out of 21 (76%) in the DP6-001 trial (Fig 3D). This data indicates that both V3 and co-receptor binding site antibodies play a role in neutralizing the sCD4-treated JR-FL virus.

**Figure 4:**
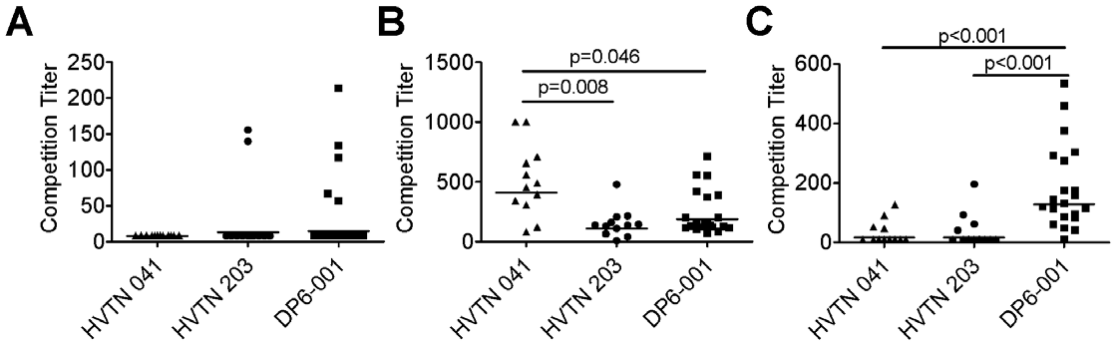
Specificity of vaccine-induced antibody responses as determined through mAb competition. The ability of serially diluted human immune serum to outcompete binding of mAb to a JR-FL & VSV-G pseudotyped virus was measured. Competition titers indicate the serum dilution preventing 50% of pseudoviral binding to the mAb. A) Competition with carbohydrate-specific mAb, 2G12. B) Competition with V3 loop-specific mAb, 447-52D. C) Competition with CD4bs-specific mAb, b12.

Competitive binding assays were conducted against known broadly neutralizing mAbs. Minimal competition was seen against the glycan-specific 2G12 mAb (Fig 4A). None of the 12 sera from the HVTN 041 trial, 2 of the 12 sera (17%) from HVTN 203, and 5 of 21 (24%) from DP6-001 outcompeted binding to 2G12. In contrast, antibodies with specificities similar to that of the V3-specific mAb, 447-52D, were elicited nearly ubiquitously in all of the vaccinee sera tested (Fig 4B). The geometric mean competitive binding titers against 447-52D were 1∶108 for the HVTN 203 sera, 1∶409 for HVTN 041, and 1∶187 for DP6-001. Statistically significant differences in the titers of V3-directed antibodies were observed in the HVTN 041 sera relative to the HVTN 203 sera (p = 0.008) and DP6-001 sera (p = 0.046).

A unique profile of CD4bs-directed antibodies was observed upon examination of the ability of the immune sera to outcompete binding against mAb b12 (Fig 4C). Only 4 out of 12 sera (33%) from either the HVTN 203 trial or HVTN 041 generated an antibody response capable of outcompeting binding to b12. However, 20 out of 21 sera (95%) from the DP6-001 trial were capable of outcompeting binding to b12 and did so with significantly higher titers, sometimes exceeding a 1∶500 dilution (p<0.001 against both HVTN 041 and HVTN 203 sera).

**Figure 5:**
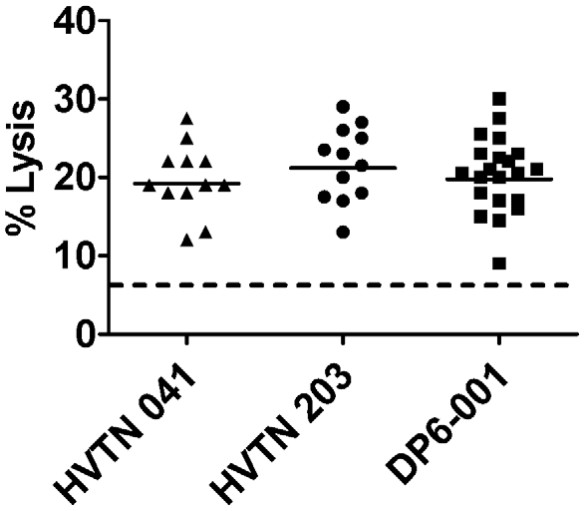
Ability of vaccinee sera to mediate ADCC activity. CEMNK^r^ target cells were pulsed with gp120 prior to exposure of vaccine serum at a 1∶100 dilution. Target cell lysis indicates the ability of vaccinee serum to mediate cell killing by PBMC from a normal human donor. Dotted line indicates background cell lysis observed with a normal human sera control.

**Figure 6:**
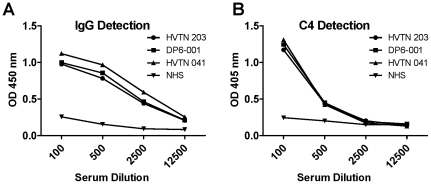
The ability of Env-specific antibodies to activate the complement cascade present in complement intact normal human sera was determined using deposition of C4 as a marker for complement activation. A representative plot with data from a single individual from each trial is shown. A) gp120-specific IgG measurement and B) C4 detection in the same testing sera.

Additional functions of gp120-specific antibodies were analyzed. Immune sera elicited by all three vaccine regimens were capable of mediating ADCC function in an equivalent fashion with 19–21% lysis of the recombinant gp120 protein pulsed CEMNK^r^ target cells (Fig. 5). An additional intrinsic characteristic of antigen-specific antibody is the ability to mediate activation of the complement pathway. Complement activation by gp120-specific antibody was conducted for sera from all three trials; however, they all activated complement in a similar fashion. A representative assay result is shown in Fig. 6A–B. A summary of the antibody profiles for each set of immune sera analyzed in the current study is provided (Table 2).

**Table 2:** Profiles of antibody responses elicited by three HIV vaccine regimens.

	HVTN 041 (Protein alone)	HVTN 203 (Viral vector + protein)	DP6-001 (DNA + protein)
NAb against sensitive primary virus	High titer NAb	Low titer NAb	High titer NAb
NAb against less resistant primary virus	Positive NAb with moderate frequency	Occasionally positive NAb	Positive NAb with moderate frequency
NAb against more resistant primary virus	Occasionally positive NAb	No detectable NAb	Positive NAb with moderate frequency
Serum gp120-specific binding IgG	Significantly high level binding antibodies	High level binding antibodies	Moderately high level binding antibodies
Ab against CD4i	Positive	Positive	Positive
Ab competing mAb 2G12	Undetectable	Rare	Occasional
Ab competing mAb 447-52D	Significantly high level Ab	Moderately high level Ab	High level Ab
Ab competing mAb b12	Occasional	Occasional	Frequent
ADCC activity	Positive	Positive	Positive
Complement activation	Easily detected	Easily detected	Easily detected

## Discussion

In the current report, a side-by-side comparison was conducted on the quality of human antibody responses elicited by three candidate AIDS vaccines focusing on HIV-1 Env-specific antibodies. Vaccines from all three studies had a gp120 protein vaccine component but only two of the studies included priming immunizations using either a viral vector- or DNA-based vaccine. Although the sample sizes are relatively small, our results suggest that antibody profiles elicited by each vaccination regimen are different. This information is valuable for the development of AIDS vaccines with potential to elicit protective antibody responses.

It has been shown in many published studies that passive transfusion of antibodies in non-human primate models can provide protection against challenge [4], [5], [6], [7], [8], [9], [10], but the real challenge is that there is limited information on the antibody specificities which may have contributed to such protection. This knowledge is important because it may contribute to the design of more effective vaccine antigens.

All three vaccines generated a high titer binding antibody response. HVTN trial 041 volunteers had the highest Env-specific serum IgG titers. Previous studies using recombinant gp120 proteins alone adjuvanted in alum did not generate high binding antibodies [13]. Therefore, it is very likely that the strong adjuvant effect of AS02_A_ (MPL and QS21 formulated in o/w emulsion) played an important role in the high immunogenicity observed for this gp120 protein-based vaccine.

The levels of binding antibody did not correlate with the presence or titer of neutralizing antibodies against a panel of heterologous isolates. Consistent with previous reports [12], [14], [15], the sera from HVTN 041 and HVTN 203 trials were, for the most part, only capable of neutralizing sensitive isolates of HIV-1, whereas sera from the DP6-001 displayed neutralization activities against more resistant isolates.

In an attempt to explain this disparity, the fine specificity of antibodies elicited by each trial was characterized. While antibodies specific for glycan, CD4i, and V3 loop epitopes on gp120 were all found at relatively similar levels among three sets of immune sera, antibodies specific for the CD4bs were found more frequently and in significantly higher titer in the DP6-001 study. This may be important as CD4bs antibodies were found responsible for the broad neutralizing activities in HIV-infected patients [22].

Very few differences were observed when other biological functions, such as complement activation and ADCC activity, were determined. This somewhat contradicts previous data indicating that the canarypox prime–recombinant Env protein boost was more effective in eliciting higher levels of binding antibody and higher frequencies of ADCC responses than the same recombinant Env when used alone [23], [24]. However, the immunogenicity of recombinant Env proteins used in those studies was clearly less optimal as shown by low levels of binding antibodies to the V3 loop. The reason for this difference is not entirely clear but a more immunogenic recombinant Env formulation with a potent adjuvant system may be responsible for the high binding titers and high frequency of ADCC observed in HVTN 041 sera. However, this finding does not change the fact that canarypox prime–recombinant Env boost is also highly immunogenic although it is not more effective in generating ADCC than recombinant protein vaccines when optimally formulated.

Sera from HVTN 203 had the least unique antibody profile. It is less effective than DP6-001 sera in eliciting conformationally-sensitive antibodies and neutralizing activity, and less effective at raising binding antibody responses than HVTN 041 sera. It is not clear whether these differences between the canarypox vector prime and the DNA vaccine prime can be attributed to the fact that the canarypox vector expresses multiple unrelated viral vector proteins in addition to the HIV-1 Env while priming with the DNA vaccine only focuses on the expression of Env.

Since the same canarypox prime-recombinant Env protein boost approach was used in the recent RV144 trial which showed statistically significant protection against HIV-1 in an efficacy field trial, the results presented in the current report raise several interesting questions. If a canarypox prime-recombinant Env protein boost approach indeed offers any unique protective benefit over the other two approaches, it is then necessary to identify new biomarkers other than those included in the current study since none stood out as a unique marker for the success of the HVTN 203 trial vaccine. Alternatively, either of the two other HIV vaccines evaluated in the current study may have the potential to provide even better protection than the canarypox prime-recombinant Env protein boost approach if the higher responses in certain assays observed only in the HVTN 041 or DP6-001 trial sera are any indication. Additional late phase clinical studies are needed to answer these questions. However, since the current report showed that each vaccination approach has a relatively specific antibody response profile, it may become feasible to start linking the efficacy of any future vaccine formulation to the antibody profile it exhibits.

The current report also pointed to a great need to expand the scope of research to include diverse types of antibody responses when a candidate HIV vaccine is evaluated. The presence of neutralizing antibodies has been used almost exclusively to judge the protective potential of vaccine-induced antibody responses. Other parameters, especially the induction of conformation-dependent antibodies, can provide unique insight to differentiate the quality of antibodies elicited by vaccines.

In recent studies of HIV-infected individuals with broadly neutralizing activity, the neutralizing fraction of sera has often been mapped to those antibodies directed towards the CD4bs [22], [25], [26], [27]. Several new broadly neutralizing mAbs were developed targeting the CD4bs [28]. Two other new mAbs, PG9 and PG16, also target at conformationally sensitive epitopes which were formed by domains from different adjacent gp120 antigens [29]. Other non-HIV recombinant protein-based vaccines, such as HBV and HPV vaccines, also require highly conformational antigens [30], [31], [32]. Because of this, it is exciting to observe the elicitation of antibodies against conformationally sensitive CD4bs as those seen in HIV-infected individuals through the use of a DNA prime-protein boost regimen. The unique antibody profile and ability to better neutralize primary isolates provides evidence that the DNA prime-protein boost regimen offers another promising heterologous prime-boost platform for further HIV vaccine development in addition to the recent RV144 canarypox prime-protein boost regimen.

## Materials and Methods

### HIV vaccine trial vaccinee sera

Human serum samples from the HVTN 041 (NCT00027365) and 203 (NCT00007332) trials [12], [14] were obtained through an ancillary study agreement with the US NIH HIV Vaccine Trials Network (HVTN). Sera from DP6-001 study (NCT00061243) were collected as previously described [15]. All serum samples used in this study were collected two weeks after the final immunization.

### Ethics Statement

Human serum samples used in the current study were provided by previously closed human clinical trials. The samples used for the current analysis do not have any identifying information about the volunteers that were included in the original studies. Two of these previously closed studies, HTVN trials (HVTN 203 (NCT00007332) and HVTN 041 (NCT00027365)), were conducted by US National Institute of Health' HIV Vaccine Trial Network (HVTN). The Institutional Review Board (IRB) of each participating site of these trials reviewed and approved these study protocols and informed consent forms according to ethics requirements established by HVTN. For the DP6-001 study (NCT00061243), study protocol and informed consent were reviewed and approved by IRB at the University of Massachusetts Medical School (UMMS), Worcester, MA, USA. For each study, IRB approved written consent was obtained from all study participants. UMMS IRB has reviewed current study of serum analysis and waived requirement of informed consent since these sera were unused samples from previously closed studies without any volunteer identifier information.

### Antibodies

Monoclonal antibodies used in the current study were provided by Drs. Dennis Burton (b12), Susan Zolla-Pazner (447-52D), and James Robinson (17b), and NIH AIDS Research & Reference Reagent Program (2G12).

### Cells and Cell Lines

TZM-bl and CEMNK^r^ cells were obtained from the NIH AIDS Research and Reference Reagent program. PBMC used as effector cells in the ADCC assays were obtained from Dr. Marjorie Robert-Guroff.

### Enzyme-linked Immunosorbent Assay

Endpoint binding titers were determined by applying serially diluting serum samples from each trial to JR-FL gp120-coated microtiter plates at 1 µg/mL. Bound gp120-specific IgG was detected using a biotinylated anti-human antibody and a subsequent incubation with a streptavidin-HRP. After development with a 3,3′5,5′-tetramethylbenzidine substrate solution, endpoint titers were defined as the last dilution of sera providing at least twice the background optical density of a normal human sera control.

### Neutralization Assays

Neutralization assays were done as previously described [33]. Briefly, 200 TCID_50_ was incubated with human sera for 1 hr, followed by the addition of 10^5^ TZM-bl cells in a final concentration of 20 µg/mL DEAE Dextran. Plates were incubated at 37°C for 48 hours and developed with luciferase reagent (Promega). Neutralization was calculated as the percent change in luciferase activity in the presence of normal human sera versus that of luciferase activity in the presence of immune sera [(NHS RLUs – Immune RLUs)/(NHS RLUs)]*100. In some neutralization assays, JR-FL pseudovirus was treated with 5 µg/mL sCD4 for 1 hr at 37°C prior to the addition of serum. When peptide adsorptions were reported, serum was incubated with a consensus clade B V3 peptide (CTRPNNNTRKSIHIGPGRAFYTTGEIIGDIRQAHC) at 25 µg/mL for 1 hr at 37°C prior to the addition of virus.

### Competitive Binding Assays

Competitive binding assays were performed as previously described [34], [35] with minor modifications. Pseudovirions bearing the JR-FL Env and Vesicular Stomatitis Virus were incubated with serial dilutions of human vaccinee sera prior to the addition to a mAb-coated microtiter plate. Virus/sera mixture was then incubated in the ELISA wells for 3 hrs at room temperature. Plates were washed and 10,000 TZM-bl cells per well were overlayed and incubated for 48 hrs at 37°C. Competition activity is reported as the serum dilution at which the luciferase signal is reduced by 50%.

### ADCC

The ability of serum from immunized individuals to mediate ADCC activity was performed as previously described with minor modifications [36]: 1×10^6^ CEMNK^r^ cells were dual stained with 2.5×10^−6^ M PKH-26 (Sigma) and 5×10^−8^ M CFSE (Molecular Probes, Invitrogen). The labeled cells were pulsed with 5 µg gp120, and exposed to vaccine sera prior to incubation with PBMC from an HIV negative donor for 4 hours. Cells were then subjected to flow cytometric analysis where CEMNK^r^ target cell lysis was defined as the percentage of CEMNK^r^ cells in the PKH-26^hi^ population that lost CFSE fluorescence.

### Detection of complement activation

The downstream product of complement activation, C4, was detected in an ELISA-based assay. ELISAs were performed as described above, where JR-FL gp120 protein was coated on a microtiter plate and exposed to serial dilutions of heat inactivated vaccinee sera. After washing, intact normal human serum was used as a source of complement and was incubated on the plate at a 1∶100 dilution for 1 hr at RT. Deposited C4 was then detected with a goat anti-C4 antibody (1∶1000 dilution for 1 hr incubation at RT). An AP conjugated anti-goat secondary antibody was used for final detection.
